# Comparison of continuous pericapsular nerve group (PENG) block versus continuous fascia iliaca compartment block on pain management and quadriceps muscle strength after total hip arthroplasty: a prospective, randomized controlled study

**DOI:** 10.1186/s12871-023-02190-1

**Published:** 2023-07-11

**Authors:** Lei Duan, Liang Zhang, Chuang-Guo Shi, Li-Gang Huang, Hui Ao, Ze-Peng Wang, Yue Deng, Meng-Liang Sun

**Affiliations:** 1 Department of Anesthesiology, Xi’an Aerospace General Hospital, Xi’an, China; 2 Department of Joint Surgery, Xi ’an Aerospace General Hospital, Xi’an, China; 3 Intensive Care Medicine Center of Xi’an People’s Hospital, No. 155, Cross of East Aero-East Road and Hangkai Road, Chang’an District, Xi’an City, 710100 Shaanxi Province China

**Keywords:** Continuous pericapsular nerve group (PENG) block, Continuous fascia iliac compartment block (FICB), Postoperative analgesia, Total hip arthroplasty, Clinical study

## Abstract

**Background:**

This investigation aimed to evaluate the impact of continuous pericapsular nerve group (PENG) block and continuous fascia iliac compartment block (FICB) on postoperative pain following total hip arthroplasty (THA).

**Methods:**

This prospective, randomized, and controlled trial recruited a cohort of fifty-seven patients with unilateral femoral neck fractures from Xi’an Aerospace General Hospital in northwest China between July 2020 and November 2021. These patients were randomly assigned to two groups: the continuous PENG block group (PENG group, n = 29) and the continuous FICB group (FICB group, n = 28). Under ultrasound guidance, PENG block and FICB procedures were performed prior to spinal anesthesia, utilizing 20 ml of 0.25% ropivacaine for PENG block and 30 ml of 0.25% ropivacaine for FICB. Subsequently, a catheter was inserted. All study participants received a standardized postoperative multimodal analgesic regimen, including intravenous administration of 30 mg Ketorolac tromethamine every eight hours and patient-controlled neural analgesia (PCNA) after surgery. Numerical rating scale (NRS) scores at rest and during exercise were recorded at various time points: prior to block (T0), 30 min post-blockade (T1), and 6 h (T2), 12 h (T3), 24 h (T4), and 48 h (T5) postoperatively. Additional data collected encompassed postoperative quadriceps muscle strength, the time of initial ambulation after surgery, the number of effective PCNA activations, rescue analgesia requirements, and occurrences of adverse events (such as nausea and vomiting, hematoma, infection, catheter detachment, or displacement) within 48 h following surgery.

**Results:**

In the PENG group, the resting NRS pain scores exhibited lower values at T1, T4, and T5 than those at T0. Furthermore, exercise NRS pain scores at T1-T5 were lower in the PENG group than in the FICB group. Similarly, during the same postoperative period, the PENG group demonstrated enhanced quadriceps strength on the affected side compared to the FICB group. Additionally, the PENG group displayed earlier postoperative ambulation and reduced occurrences of effective PCNA activations and rescue analgesia requirements compared to the FICB group.

**Conclusion:**

Continuous PENG block exhibited superior analgesic efficacy after THA compared to continuous FICB, promoting recovery of quadriceps strength on the affected side and facilitating early postoperative ambulation.

**Trial Registration:**

This clinical trial was registered in the China Clinical Trials Center (http://www.chictr.org.cn) on 20/07/2020, with the registration number ChiCTR2000034821.

## Introduction

The hip joint, a crucial ball-and-socket joint essential for lower limb mobility [1], is susceptible to femoral neck fractures (FNF), which occur below the femoral head and above the base of the femoral neck due to direct or indirect force. FNF has been identified as a major cause of mobility impairment in older people [2]. Elderly patients with FNF are prone to complications such as femoral head necrosis and fracture non-union, significantly impacting their daily life and overall health. Consequently, FNF has become a significant societal concern, with projections indicating a rise in hip fracture incidence from 4.5 million in 2019 to 21.3 million in 2050 as the global population ages, with the Asian population accounting for approximately 45% of cases [3, 4].

Total hip arthroplasty (THA) is a commonly employed procedure for FNF treatment. However, severe postoperative pain following THA delays motor function recovery, increases the risk of hospitalization and thromboembolic events, and adversely affects long-term patient outcomes [5, 6]. Therefore, there is a need for improved perioperative analgesia to alleviate patient pain and promote postoperative recovery [7, 8]. Opioids and regional blocks are the primary modalities for managing acute pain after THA, as they significantly reduce postoperative pain [9, 10]. Nonetheless, the use of potent opioids is constrained due to their inherent side effects, including nausea, vomiting, and respiratory depression. While fascia iliac compartment block (FICB) is a commonly used regional block technique for THA, it presents challenges in blocking the obturator nerve and may impact the femoral nerve, leading to inadequate postoperative analgesia and an increased risk of falls [11, 12]. Conversely, pericapsular nerve group (PENG) block, a novel targeted block technique for the sensory branch of the anterior hip capsule, achieves the desired analgesic effect without affecting muscle strength, thus facilitating postoperative functional recovery [13, 14]. Notably, the PENG block offers the advantage of being performed in the supine position, which is particularly beneficial for patients with chronic pain or acute hip fractures [15]. However, a single PENG block provides only short-term effectiveness and does not offer continuous analgesia. There have been no reports on the effectiveness of continuous PENG block for postoperative analgesia after THA.

This study aims to compare the analgesic efficacy and safety of continuous PENG block with continuous FICB during the perioperative period in elderly patients with FNF. The primary outcomes to be evaluated include the numerical rating scale (NRS) scores at rest and during exercise, recorded prior to block (T0), 30 min post-blockade (T1), and at 6 h (T2), 12 h (T3), 24 h (T4), and 48 h (T5) postoperatively. Secondary outcomes encompass postoperative quadriceps muscle strength, the time of initial ambulation after surgery, the number of effective patient-controlled neural analgesia (PCNA) activations, rescue analgesia requirements, and occurrences of adverse events (such as nausea, vomiting, hematoma, infection, catheter detachment, or displacement) within 48 h following surgery.

## Materials and methods

### Study design

**Fig. 1:**
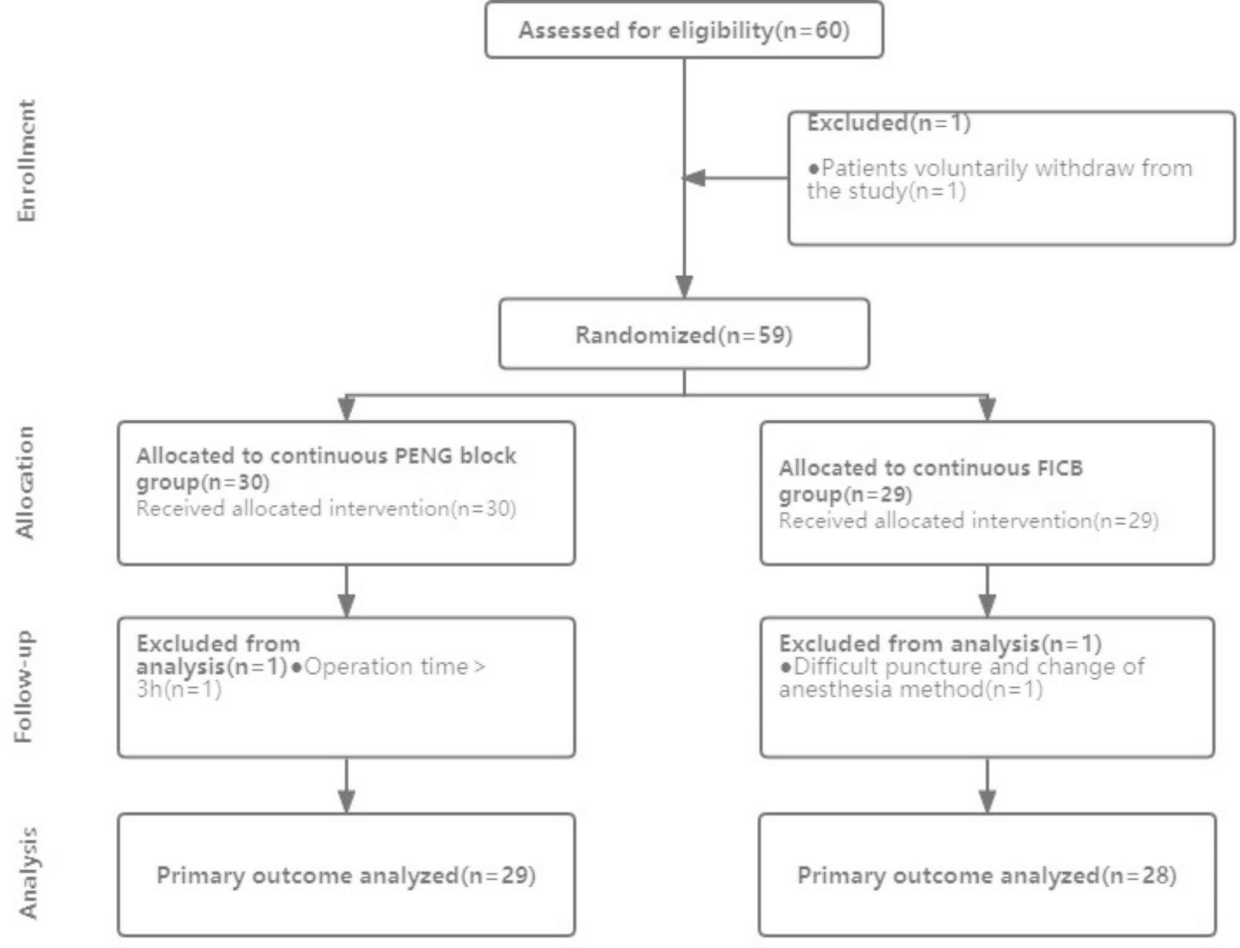
Flowchart of the entry group

This prospective, randomized, and controlled trial received approval from the Hospital Medical Ethics Committee (Lot No. XHTZYY-2020-LL-02) on 08/07/2020 and was registered with the China Clinical Trials Center (http://www.chictr.org.cn, ChiCTR2000034821) on July 20, 2020. The study adhered to the Consolidated Standards of Reporting Trials (CONSORT) statement and the declaration of Helsinki. Informed consent was obtained from all participants. The study enrolled elderly patients with femoral neck fractures scheduled for posterior-lateral approach THA under spinal anesthesia from July 2020 to November 2021 at the Department of Joint Surgery, Xi’an Aerospace General Hospital. Inclusion criteria comprised patients of either sex, aged ≥ 18 years, BMI < 35 kg/m^2^, and classified as ASA grade I-III. A total of 60 enrolled patients were randomly assigned in a 1:1 ratio to either the continuous PENG block group (PENG group) or the continuous FICB group (FICB group) using a computerized random number table method (https://www.sealedenvelope.com). Each group consisted of 30 cases. Exclusion criteria encompassed individuals with (1) cognitive dysfunction or ineffective communication, (2) contraindications to peripheral nerve block and spinal anesthesia (e.g., skin infection in the inguinal region and back or rupture at the puncture site, abnormal coagulation function or disorders), (3) abnormal sensory and motor function of the lower limbs, (4) chronic pain, history of long-term analgesic drug use, or opioid addiction, and (5) allergies to local anesthetic drugs. Additional exclusion criteria included (i) intraoperative bleeding ≥ 500 ml, (ii) operation time ≥ 3 h, (iii) conversion from spinal anesthesia to general anesthesia due to spinal anesthesia failure, and (iv) voluntary withdrawal by patients, as shown in Fig. 1.

### Grouping and interventions

Treatment allocation was concealed using consecutively numbered, sealed, opaque envelopes held by an anesthesia nurse who was not involved in the study. The envelopes were handed to the anesthesiologist performing the nerve block on the day of surgery. Both groups of patients fasted for eight hours and refrained from clear liquids for 2 h before surgery. In the preoperative holding area, all study patients received intravenous parecoxib sodium 40 mg and fentanyl 1.5 µg/kg. Upon entering the operating room, non-invasive arterial blood pressure (NIBP), heart rate (HR), pulse oximetry (SpO2), and three-lead electrocardiogram (ECG) were continuously monitored.

**Fig. 2:**
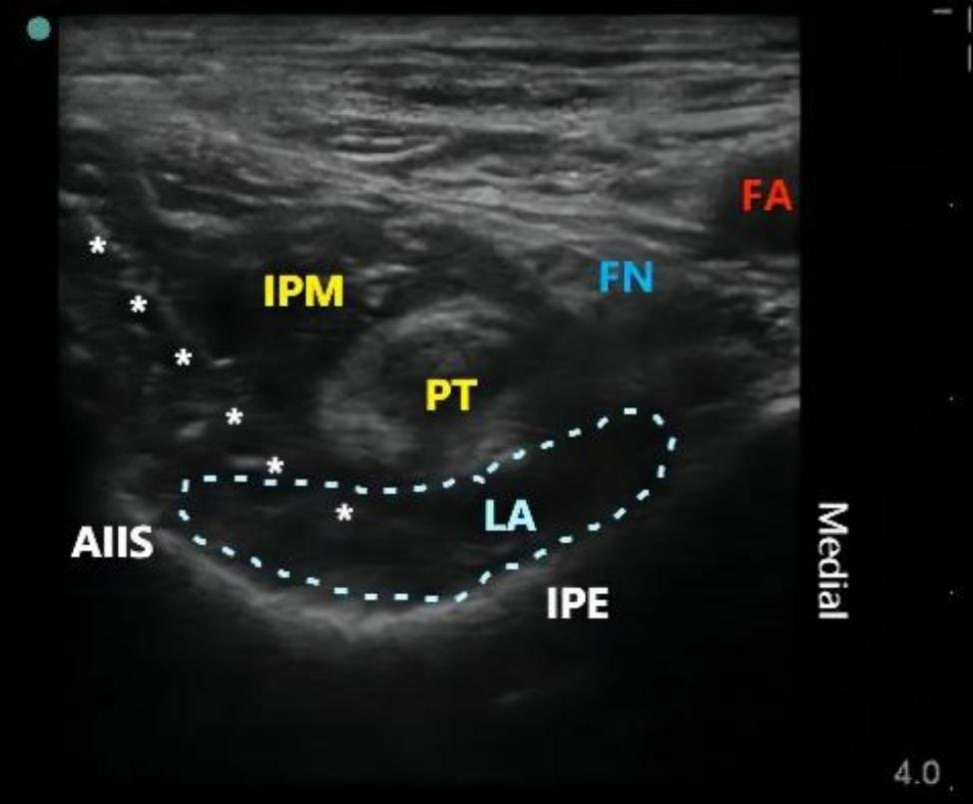
Continuous PENG block. AIIS: anterior inferior iliac spine; white asterisk: catheter trajectory; IPE: iliopubic eminence; LA: local anesthetic; PT: psoas major tendon; IM: iliacus muscle; white triangle: iliac Fascia; FA: femoral artery

The procedure in the PENG group [16]: the anterior superior iliac spine (ASIS) location on the affected side was identified in the supine position. After routine skin disinfection, a low-frequency convex array probe (frequency 2–5 MHz, S-series, Sonosite, USA) was placed horizontally on the edge of the ASIS plane and then rotated 45° caudally to align with the pubic branch. The AIIS was visualized through the acoustic window, followed by the caudal movement of the probe to identify the location of the iliopubic eminence (IPE). The psoas tendon (PT) was identified as the highlighted oval structure above the IPE. Using an in-plane technique, a puncture needle (18G×100 mm, Contiplex type D, Braun, Germany) was inserted from lateral to medial, between the IPE and the PT, ensuring no blood or gas was withdrawn. Subsequently, 20 ml of 0.25% ropivacaine was injected, and the drug spread in a low-echo shuttle shape between the PT and the IPE. Following this, a catheter (20G×120 mm, single hole, Braun, Germany) was inserted deep into the PT (Fig. 2)at a depth of 5 cm. An ultrasound scan was conducted to verify the proper fixation of the catheter on the inguinal ligament, thereby preventing interference with the surgical incision area.

**Fig. 3:**
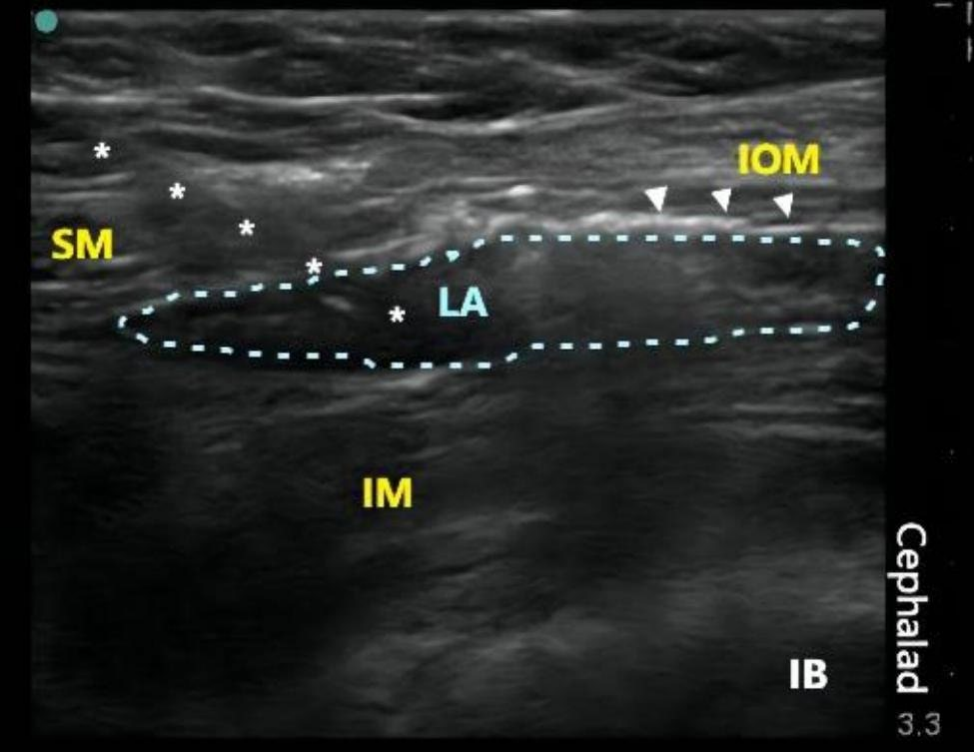
Continuous FICB. SM: sartorius muscle; white asterisk: catheter trajectory; LA: local anesthetic; IM: iliacus muscle; IB: iliac bone; white triangle: iliac Fascia; IOM: internal oblique muscle

The procedural technique employed in the FICB group followed a previously conducted study [17]. The patient assumed a supine position to ascertain the location of the ASIS on the affected side. Following routine disinfection of the skin, a high-frequency line array probe (frequency 6–13 MHz, S-series, Sonosite, USA) was positioned vertically on the inguinal ligament, specifically in the outer one-third of the line between the pubic tubercle and the ASIS. The sonographic examination identified a “bow-tie sign” resembling structure in the region above the anterior inferior spine, corresponding to the Iliofascial gap interval. The narrowest point of the “bow-tie” sign represents the convergence of the iliac fascia and the fascia lata, with the obliquus internus abdominis muscle located on the cranial side and the sartorius muscleon the caudal side. The fascia iliac (FI), characterized by a high echogenic bright line above the iliopsoas muscle, was visualized. From a caudal to a cephalic plane, an 18G×100 mm puncture needle (Contiplex D, Braun, Germany) was inserted into the deep surface of the FI. A hydrodissection technique using 3 ml of normal saline was employed to confirm a bloodless and gas-free tip position. Subsequently, 30 mL of 0.25% ropivacaine was injected, and the drug diffused below the FI in a low-echo shuttle shape. The catheter (20G×120 mm, single hole, Braun, Germany) was then placed at a depth of 5 cm within the FI, as depicted in Fig. 3. An additional ultrasound scan was conducted to ensure proper fixation of the catheter on the inguinal ligament, thereby preventing interference with the surgical incision area.

### Anesthesia application

Thirty minutes after completion of the block, the patient was repositioned in the healthy side-lying position, with the affected side facing upward, and spinal anesthesia was performed at the L3-4 interspace. Upon penetration of the spinal anesthesia puncture needle into the arachnoid and subsequent cerebrospinal fluid outflow, 2.5-3.0 ml of 0.25% bupivacaine was slowly administered to maintain the sensory block level at T10. Blood pressure (BP) and heart rate (HR) were maintained within 20% of the basal value during the operation.

In both groups, local infiltration anesthesia with 10 ml of 0.25% ropivacaine was performed at the incision site before the conclusion of the surgery. Neither the PENG block nor the FICB provided a complete blockade of the surgical incision area. Therefore, a local infiltration block was performed in both groups before suturing the skin to ensure uniform analgesia in the incision area and eliminate confounding factors associated with superficial skin pain.

The same orthopedic team performed the surgical procedures, who had completed approximately 40 PENG/FICB block cases before enrolling patients. Perioperative observation parameters were assessed and recorded by the same anesthesiologist who was not involved in the study.

### Postoperative analgesia protocol and rescue analgesic

Following the surgical procedure, patients were admitted to the post-anesthesia care unit (PACU) and subsequently transferred to the ward upon stabilization of vital signs. All patients received the same postoperative multimodal analgesia regimen, which included intravenous administration of ketorolac tromethamine 30 mg every eight hours and patient-controlled neural analgesia (PCNA) after surgery. The PCNA formula consisted of 0.10% ropivacaine (300 ml), and the parameters for PCNA was set as follows: continuous infusion dose of 5 ml/h, bolus dose of 5 ml, lock time of 30 min, and continuous analgesia for 48 h. In cases where the NRS was ≥ 4 points and persisted for more than 30 min, intravenous rescue analgesia was administered using oxycodone hydrochloride at a dose of 0.1 mg/kg.

### Measurements

The numerical rating scale (NRS) at rest and during exercise were recorded as the primary outcome at multiple time points: before block (T0), 30 min post-blockade (T1), and 6 h (T2), 12 h (T3), 24 h (T4), and 48 h (T5) postoperatively. Exercise pain was evaluated based on the NRS score corresponding to passive straight leg elevation at 15° [18]. Secondary outcomes included quadriceps muscle strength assessment at 24 and 48 h postoperatively, time of the first initial ambulation after surgery, number of effective presses, rescue analgesia frequency, and incidence of adverse events at 48 h postoperatively (e.g., nausea, vomiting, hematoma or infection at the puncture site, catheter displacement).

The NRS scores were employed to evaluate the level of pain experienced by the patients, with 0 points indicating no pain, 1–3 points indicating mild pain, 4–7 points indicating moderate pain, and 8–10 points indicating severe pain. Quadriceps strength was assessed using an OE-210 hand-held dynamometer (HDD) (Ito Ultra Shortwave, Japan) at 24 and 48 h postoperatively [19]. The patient assumed a supine position with the knee flexed at 60° and attempted to straighten the knee joint with maximum force while the rehabilitation physician applied resistance by placing the probe of the HDD on the distal front of the calf. The evaluation aimed to measure the strength of the quadriceps during a maximum isometric contraction. Catheter displacement was assessed by injecting 1 ml of saline at the catheter’s end and conducting an ultrasound scan of the catheter tip every 24 h after surgery. Displacement was considered to have occurred if the catheter tip had moved outward by ≥ 5 cm.

### Statistical analysis

The Kolmogorov-Smirnov normality test was conducted for continuous variables. Normally distributed variables were expressed as mean ± standard deviation (SD), and independent samples t-test was used for between-group comparisons. Non-normally distributed variables were expressed as median (interquartile range, IQR) [M (Q1, Q3)], and the Mann-Whitney U test was employed for between-group comparisons. Repeated-measures ANOVA was utilized to compare multiple time points. Count data were presented as the number of cases, and the Chi-square test or Fisher’s exact probability analysis was conducted for between-group comparisons. The significance level was set at α = 0.05, and differences were considered statistically significant at P < 0.05.

The sample size calculation was based on a pilot study that included eight patients in each group using PASS 11 software. The calculation was performed based on the 24-hour postoperative exercise NRS scores in both groups (3 [2, 3] in the continuous PENG group and 4 [3, 4] in the continuous FICB group), with α = 0.05 and 1-β = 0.90. Consequently, 25 cases were required in each group, accounting for a 10% loss of visit rate. Therefore, this study aimed to enroll 30 patients in each group.

## Results

### General information

A total of 60 patients were initially recruited for the study, with one patient excluded from the PENG group due to the operation duration exceeding 3 h. Ultimately, the statistical analysis included 29 cases in the PENG group. Similarly, one patient was excluded from the FICB group due to lumbar puncture difficulty, and an additional patient withdrew voluntarily, resulting in 28 cases included in the statistical analysis for the FICB group. There were no significant differences in age, gender, BMI, ASA classification, surgical time, and intraoperative bleeding between the two groups (all P > 0.05). The detailed data are shown in Table 1.

**Table 1:** Comparison of the general conditions of patients in the two groups

	PENG group, n = 29	FICB group, n = 28	*P*_value_
Age (years)	68.3 ± 6.3	69.0 ± 7.2	0.676
Sex, F/M	13/16	14/14	0.696
BMI (kg/m^2^)	23.8 ± 3.5	24.3 ± 3.6	0.603
ASA physical status,I/II/III	4/16/9	3/15/10	1.000^*^
Duration of surgery (min)	103.2 ± 17.8	101.4 ± 15.1	0.683
Intraoperative bleeding (ml)	231.3 ± 57.4	240.0 ± 49.5	0.565

Data are presented as mean ± SD or number of patients.^*^P value for the χ^2^ test. PENG group: continuous pericapsular nerve group block; FICB group: continuous fascia iliac compartment block. BMI: body weight index. ASA: American Society of Anesthesiologists

### Comparison of resting NRS Pain Scores

**Fig. 4:**
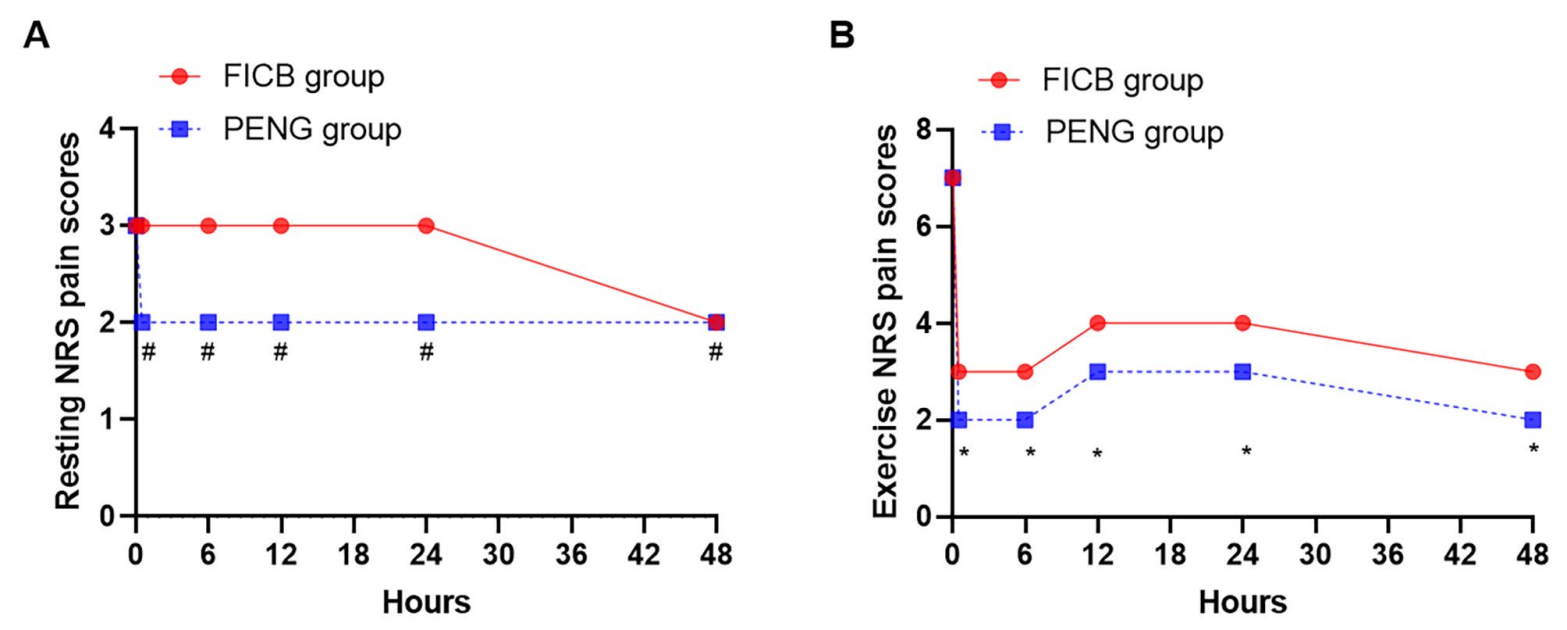
Trends in resting (**A**) and exercise (**B**) NRS pain scores of the two groups. Note: P < 0.05 compared with the FICB group, label∗; P > 0.05 compared with the FICB group, label#. PENG group, continuous pericapsular nerve group block; FICB group, continuous Fascia iliac compartment block. NRS, numerical rating scale

**Fig. 5:**
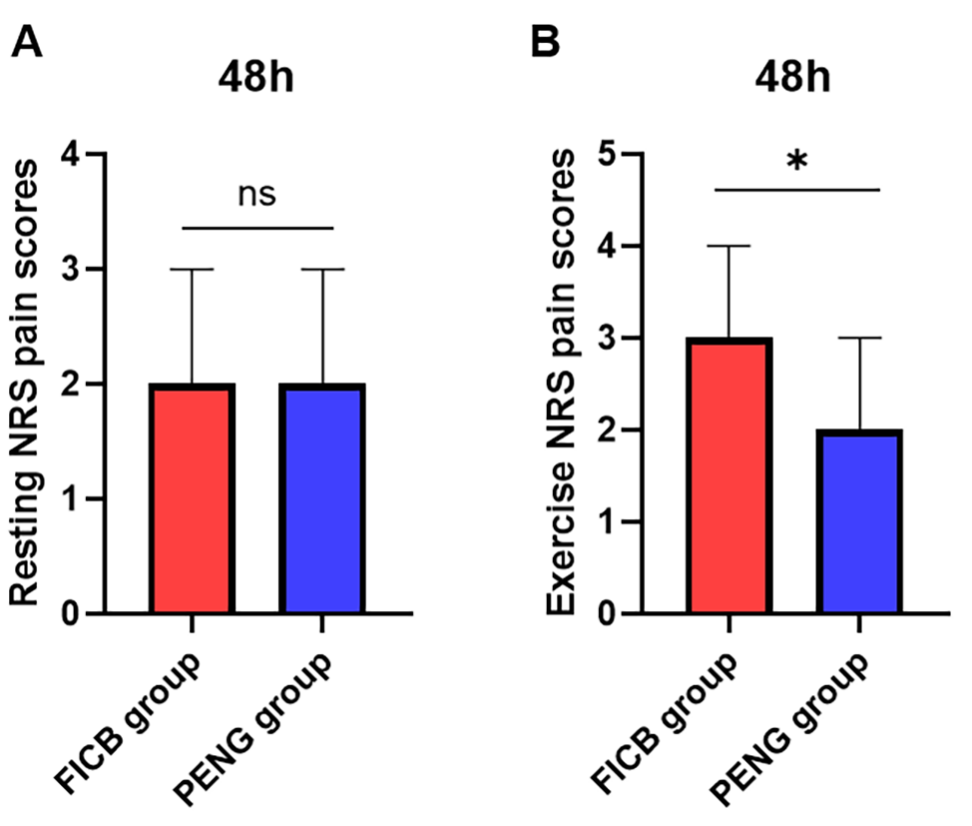
Resting (**A**) and exercise (**B**) NRS pain scores at T5 (48 h after surgery) of the two groups. Note: ∗, P < 0.05 compared with the FICB group; ns, P > 0.05 compared with the FICB group. PENG group, continuous pericapsular nerve group block; FICB group, continuous Fascia iliac compartment block. NRS, numerical rating scale

The overall analysis demonstrated a statistically significant difference (P < 0.05) in resting NRS pain scores between the two groups at different times. In pairwise comparisons within the PENG group, T1, T4, and T5 showed lower resting NRS pain scores than T0, with statistically significant differences (P < 0.05). However, within the FICB group, no statistically significant differences existed in resting NRS pain scores during the same period (P > 0.05). The difference in resting NRS pain scores between the two groups was insignificant (P > 0.05). The detailed data are shown in Table 2; Fig. 4, and Fig. 5.

**Table 2:** Comparison of resting NRS pain scores between the two groups

	PENG group, n = 29	FICB group, n = 28	*F*_intergroup,_ *P*	*F*_time,_ *P*	*F*_Interaction,_ *P*	PENG group vs. FICB group median difference (95% CI), *P*
T0	3 (1, 4)	3 (2, 4)	3.149, 0.087	18.661, < 0.001	1.662, 0.148	-1 (-1 to 0), 0.084
T1	2 (1, 3) ^a^	3 (1, 4)				0 (-1 to 0), 0.063
T2	2 (2, 3)	3 (2, 3)				0 (-1 to 0), 0.101
T3	2 (2, 3)	3 (2, 3)				0(-1 to 0), 0.115
T4	2 (1, 3) ^a^	3 (1, 3)				0 (-1 to 0), 0.171
T5	2 (1, 3) ^a^	2 (1, 3)				0 (-1 to 0), 0.284

Data are presented as median (IQR). Multiple time points were compared using repeated-measures ANOVA. Comparison between the groups was analyzed using the Mann-Whitney U test. *F*_intergroup_: *F*-value of the test statistic for inter-group comparison between groups; *F*_time_: *F*-value of the test statistic for multiple time points comparison between groups; *F*_Interaction_: *F*-value of the test statistic for interaction comparison between groups. ^a^ Denotes statistical significance compared to the T0 within the group. PENG group: continuous pericapsular nerve group block; FICB group: continuous fascia iliac compartment block. NRS: numerical rating scale. CI: interquartile range

**Comparison of Exercise NRS Pain Scores** The overall analysis revealed statistically significant differences (P < 0.05) in exercise NRS pain scores between the two groups when comparing groups, time, and interaction. In pairwise comparisons between groups, the PENG group had lower exercise NRS pain scores than the FICB group at T1-T5, with statistically significant differences (P < 0.05). However, at T0, the two groups had no significant difference in exercise NRS pain scores (P > 0.05). In within-group comparisons, both groups showed lower exercise NRS pain scores at T1-T5 compared to T0, with statistically significant differences (P < 0.05). The detailed data are shown in Table 3; Fig. 4, and Fig. 5.

**Table 3:** Comparison of exercise NRS pain scores between the two groups

	PENG group, n = 29	FICB group, n = 28	*F*_intergroup,_ *P*	*F*_time,_ *P*	*F*_Interaction,_ *P*	PENG group vs.FICB group median difference (95% CI), *P*
T0	7 (6, 8)	7 (6, 8)	16.931, < 0.001	322.145, < 0.001	3.930, 0.002	0 (-1 to 0), 0.870
T1	2 (1, 3) ^ab^	3 (2, 4) ^a^				-1 (-1 to 0), 0.003
T2	2 (2, 3) ^ab^	3 (3, 4) ^a^				-1 (-2 to -1), < 0.001
T3	3 (3, 4) ^ab^	4 (3, 5) ^a^				-1 (-1 to 0), 0.001
T4	3 (2, 3) ^ab^	4 (3, 4) ^a^				-1 (-1 to -1), < 0.001
T5	2 (2, 3) ^ab^	3 (3, 4) ^a^				-1 (-1 to 0), < 0.001

Data are presented as median (IQR). Multiple time points were compared using repeated-measures ANOVA. Comparison between the groups was analyzed using the Mann-Whitney U test. *F*_intergroup_: *F*-value of the test statistic for inter-group comparison between groups; *F*_time_: *F*-value of the test statistic for multiple time points comparison between groups; *F*_Interaction_: *F*-value of the test statistic for interaction comparison between groups. ^a^ Denotes statistical significance compared to the T0 within the group. ^b^ Denotes statistical significance between the groups. PENG group: continuous pericapsular nerve group block; FICB group: continuous fascia iliac compartment block. NRS: numerical rating scale.CI: interquartile range

### Comparison of postoperative motor ability and postoperative analgesia

When comparing quadriceps strength on the affected side during the same postoperative period, the PENG group exhibited higher strength than the FICB group. Furthermore, the quadriceps strength on the affected side in the FICB group was lower than that of the healthy side in the same group at 24 and 48 h postoperatively, with statistically significant differences (P < 0.05). Additionally, the PENG group had an earlier time of initial ambulation after surgery than the FICB group. The detailed data are shown in Table 4.

**Table 4:** Comparison of motor ability between the two groups after surgery

	PENG group, n = 29	FICB group, n = 28	*P*_value_
Quadriceps muscle strength (kg)			
Healthy side	8.9 ± 1.1	9.1 ± 0.9	0.422
Affected side 24 h postoperatively	8.6 ± 0.9^b^	8.2 ± 0.7 ^a^	< 0.001
Affected side 48 h postoperatively	8.7 ± 0.8^b^	8.5 ± 0.5 ^a^	< 0.001
Time of initial ambulation after surgery (hours)	11.3 ± 2.3^b^	18.7 ± 1.5	< 0.001

Data are presented as mean ± SD. ^a^ Denotes statistical significance compared to the quadriceps muscle strength of the healthy side within the group. ^b^ Denotes statistical significance between the groups. PENG group, continuous pericapsular nerve group block; FICB group, continuous fascia iliac compartment block

When comparing the number of effective PCNA presses during the same postoperative period, the PENG group had a lower number than the FICB group. Moreover, the number of rescue analgesia administrations from 0 to 24 h after surgery was lower in the PENG group compared to the FICB group, with a statistically significant difference (P < 0.05). The detailed data are shown in Table 5.

**Table 5:** Comparison of postoperative analgesia between the two groups after surgery

	PENG group, n = 29	FICB group, n = 28	*P*_value_
PCNA effective press (times)			
0-24 h postoperatively	6.5 ± 3.0^b^	10.8 ± 3.6	< 0.001
24-48 h postoperatively	2.9 ± 1.0^b^	4.6 ± 2.2	< 0.001
Rescue analgesia (times)			
0-24 h postoperatively	0^b*^	5	0.023
24-48 h postoperatively	0	0	/

Data are presented as mean ± SD or number (times). ^b^ Denotes statistical significance between the groups. ^*^P value for the Fisher exact test. PENG group = continuous pericapsular nerve group block; FICB group = continuous fascia iliac compartment block

### Comparison of adverse reactions

No nausea and vomiting occurred in the PENG group, but 3 cases occurred in the FICB group, and the difference was not statistically significant (P > 0.05). Hematoma, or infection at the puncture site, was observed between the two groups. Additionally, there were no instances of catheter displacement after surgery.

## Discussion

In this study, a total of 57 patients with femoral neck fractures were randomly assigned to receive either continuous pericapsular nerve group (PENG) block (PENG group, n = 29) or continuous fascia iliaca compartment block (FICB group, n = 28). The results showed that both groups improved exercise NRS pain scores during the observation period compared to preoperative scores. However, the passive straight leg raises 15° VAS scores were consistently lower in the PENG group than in the FICB group during the same time frame. The possible reasons for this discrepancy can be analyzed as follows. Swenson et al. discovered through magnetic resonance imaging that using 30 ml of 0.25% bupivacaine for FICB did not reach the pectineus muscle effectively, failing to achieve complete obturator nerve blockade [20]. Kantakam et al. conducted a cadaveric study in which an ultrasound-guided suprainguinal fascia iliaca block was performed [21]. They found that staining the femoral nerve, lateral femoral cutaneous nerve, and obturator nerve, required a minimum effective volume (MEV90) of 62.5 ml of local anesthetic, which was significantly higher than the clinical dosage of 40 ml [17, 22, 23]. On the other hand, Mistry et al. injected 20 ml of 0.2% ropivacaine between the tendon of the psoas major muscle and the iliopubic eminence, which fully covered the anterior lateral of the hip joint capsule (i.e., the region containing nociceptive receptors of the anterior hip capsule) [24]. This technique did not cause symptoms such as hindrance in hip joint flexion or quadriceps muscle weakness, aligning with the findings of this study. Therefore, under the conditions of similar postoperative analgesic parameters and a reduced initial dose of local anesthetic, continuous PENG block provided superior pain relief for exercise pain compared to continuous FICB. Furthermore, reducing the initial dose of local anesthetic is advantageous in minimizing the incidence of local anesthetic toxicity in elderly patients.

A previous investigation has demonstrated the beneficial impact of early mobilization on postoperative recovery and length of hospital stay [25]. In the present study, to mitigate the effect of FICB on the muscular branches of the femoral nerve and preserve quadriceps strength, the ropivacaine concentration in the PCNA pump was reduced to 0.10% in both study groups, consistent with existing literature [26]. However, this adjustment resulted in higher postoperative exercise NRS pain scores in the FICB group compared to previous reports [26, 27]. This discrepancy may be attributed to the diminished analgesic efficacy of regional blockade due to the reduced concentration of local anesthetic agents [28]. Additionally, since this study employed continuous nerve analgesia, which has the potential to exert a sustained effect on the affected muscular branches of the femoral nerve, the quadriceps strength of the patients was assessed based on previous literature [19].

The findings of this investigation indicate that, in the PENG group, postoperative quadriceps strength on the affected side was slightly lower than that of the healthy side (without statistical significance). In contrast, the FICB group exhibited significantly lower quadriceps strength on the affected side than the healthy one. Furthermore, during the same period, the postoperative quadriceps strength on the affected side was significantly higher in the PENG group than in the FICB group. These outcomes are consistent with a previous study by Hao et al., which demonstrated improved quadriceps strength with PENG block in patients undergoing THA [6]. Moreover, this study expands on previous findings by revealing, for the first time, that patients in the PENG group could mobilize earlier post-surgery compared to the FICB group. This observation suggests that PENG block may facilitate early ambulation and motor function recovery, potentially reducing complications such as infection and pressure ulcers associated with prolonged bed rest, highlighting its substantial clinical significance.

The findings of this investigation revealed that the PENG group exhibited a significantly lower number of effective PCNA compressions in the postoperative 0–24 h and 24–48 h periods, as well as a lower number of rescue analgesia administrations compared to the FICB group (P < 0.05). This observation may be attributed to the challenges faced by the FICB group in achieving a complete obturator block nerve or even femoral nerve block, given the constant 5 ml/h continuous infusion dose of PCNA for the iliac fascia gap area and the diminishing effect of spinal anesthesia. Consequently, more PCNA compressions and rescue analgesia were required in the FICB group to alleviate postoperative exercise pain. These findings suggest that a higher continuous infusion dose of PCNA may be necessary to effectively manage exercise pain 24 h after THA in continuous FICB. Notably, three individuals in the FICB group experienced nausea and vomiting after receiving ketorolac tromethamine for rescue analgesia. However, no significant difference between the two groups was observed in the occurrence of nausea and vomiting. Further investigations with an expanded sample size are warranted to validate these findings. Importantly, no adverse reactions such as puncture site hematoma or catheter displacement were observed in either group after surgery, which aligns with previous research [6, 29], underscoring the safety and efficacy of continuous PENG block. Several limitations should be acknowledged in this study. Firstly, the employed procedure was the posterolateral approach for THA. However, it is worth noting that the posterolateral approach is the most commonly reported technique for THA worldwide [30], and the efficacy of continuous PENG block in the context of the anterior approach for THA requires further investigation. Secondly, this study did not address the pain originating from the innervated nerves of the posterior hip capsule. Although hip pain primarily stems from injurious receptors in the anterior hip capsule, the posterior hip capsule’s injurious receptors are innervated by branches of the sacral plexus nerve, which are not affected by either PENG block or FICB. Consequently, future studies should consider incorporating PENG block combined with sacral plexus nerve block to optimize analgesia in hip surgery and provide additional clinical options. Thirdly, the volume of local anesthetic used in the two blocks differed in this study. As the optimal volume of local anesthetic for PENG block has not been established, the results of cadaveric studies conducted by Tran and Ciftci demonstrated that 20 ml of dye adequately spread in the anterior region of the hip joint capsule, while 30 ml of dye diffused throughout the iliopsoas, vastus intermedius, and gluteus medius muscles, and the vicinity of the femoral nerve, potentially affecting the strength of the quadriceps muscle [31, 32]. Therefore, in this study, we chose to administer 20 ml of ropivacaine for PENG block. Additionally, considering that elderly patients in the northwest region of mainland China generally have a lean and frail physique, taking into account our medical center’s experience, we reduced the volume of local anesthetic for FICB to 30 ml (lower than the commonly used dose of 40 ml in foreign medical institutions) with the aim of minimizing the systemic toxicity of local anesthetics [17, 22, 23]. However, this reduction in dosage may have an impact on the analgesic efficacy in the FICB group.

## Conclusions

In conclusion, continuous hip pericapsular nerve group block can improve perioperative pain of THA, especially postoperative exercise pain. It can fully preserve quadriceps strength, which is conducive to initial ambulation, and is a more ideal analgesic modality for THA than continuous fascia iliac compartment block.

## Data Availability

The datasets used in the current study are available from the corresponding author upon reasonable request.

## Abbreviations

PENG: Pericapsular nerve group
FICB: Fascia iliac compartment block
THA: Total hip arthroplasty
NRS: Numerical rating scale
PCNA: Patient-controlled neural analgesia
FNF: Femoral neck fractures
NIBP: Non-invasive arterial blood pressure
HR: Heart rate
SpO_2_: Pulse oximetry
ECG: Electrocardiogram
CONSORT: Consolidated Standards of Reporting Trials
MEV90: 90% minimum effective volume
AIIS: Anterior inferior iliac spine
IPE: Iliopubic eminence
PT: Psoas tendon
FI: Fascia iliac
HDD: Hand-held dynamometer
